# General formulae for transforming Pearson’s r to the scale of Cohen’s d

**DOI:** 10.1177/01466216261465015

**Published:** 2026-07-02

**Authors:** Jari Metsämuuronen

**Affiliations:** 1 Turku Research Institute for Learning Analytics, University of Turku, Turku, Finland

**Keywords:** effect size, *r* effect size, Cohen’s *d*, point-biserial correlation, point-polyserial correlation

## Abstract

The traditional thresholds for product-moment correlation (*r*) usually condensed in terms of “very small,” “small,” “medium,” “large,” “very large,” and “huge” are derived on the scale of *d*. Properly transforming an estimate of *r* to the scale of Cohen’s *d* is important for qualitatively evaluating the magnitude of the *r* effect size. A proper formula exists for a transformation between *r* and *d* in the binary and dichotomous settings, but not in polytomous or continuous settings. The traditional formula for polytomous settings assumes an equal number of cases in the subpopulations and can lead to a radically misleading transformation if the group sizes are severely imbalanced. Two general formulae are derived for transformations applicable to dichotomous, polytomous, and continuous cases, regardless of the imbalance in the group sizes. Real-life examples are given which illustrate the use of the general forms and difference between the general and the traditional forms.

## Introduction

Cohen’s *d* (Cohen, 1988) has traditionally been the benchmark for the magnitude for Pearson’s *r*. An accurate transformation between *r* and *d* is available for the point-biserial correlation (*R*_*PB*_) by Cohen (1988). However, there is no corresponding transformation formula for the polytomous settings related to the point-polyserial correlation (*R*_*PP*_) or for continuous cases. Cohen suggested using a simplified formula related to the biserial correlation (*R*_*B*_): *R*_*PB*_ × 1.253 = *R*_*B*_ (Cohen, 1988, p. 82). When applied to the polytomous settings, however, this transformation yields thresholds for effect sizes (ES) that are *too high* for “small,” “medium,” and “large” (Funder & Ozer, 2016; Gignac & Szoborai, 2016). Therefore, the correspondence between *r* and *d* can be radically misleading in applied research and meta-analyses.

This article proposes two general formulae for a robust transformation between the scales of *r* and *d*. This article enlarges discussion of “generalized Cohen d” (Metsämuuronen, 2026). Metsämuuronen (2026) proposes general formulae for a similar type of transformation between the scales of coefficient eta and *d* as well as Cohen *f* and *d*.

### PMC and Effect Size

Product‒moment correlation coefficient (PMC; Bravais, 1844; Pearson, 1896) has many applications, one of which is its use as the basis for *r* effect sizes. While the square of the correlation coefficient (*r*^2^, unadjusted *R*^2^, adjusted *R*^2^, *η*^2^, partial *η*^2^, and ω^2^) is mainly used to indicate explanatory power, the correlation *r* itself is a more essential measure in the literature concerning ESs and, specifically, in the meta-analytic literature (see, e.g., Ozer, 1985).

The concept of ES refers to the quantitative measurement or estimation of the magnitude of a phenomenon of interest in a population. Typically, it is either the strength of the relationship between two variables or the difference in the means of two or more subpopulations (e.g., Cumming & Calin-Jageman, 2017; Kelley & Preacher, 2012). These quantitative measures are often described using qualitative labels such as “small,” “medium,” or “large” inherited from Cohen’s seminal works (1969, 1977, 1988) and later expanded by Sawilowsky (2009) to include “very small,” “very large,” and “huge.” Cohen himself cautioned against a rigid interpretation of these benchmarks (Cohen, 1988, p. 25), and they have since been criticized on solid grounds (see, e.g., Correll et al., 2020; Farmus et al., 2022; Funder & Ozer, 2016; Pek & Flora, 2018). In particular, both the numerical values and their verbal interpretations (e.g., “small,” “medium,” and “large”) are inherently context-dependent and may vary substantially across research domains and study designs. A classic illustration is provided by research on acetylsalicylic acid (ASA), in which the observed effect size was extremely small (approximately *r* ≈ .03). While such an effect would typically be considered trivial in fields like education or psychology, in this medical context it was interpreted as sufficiently important to correspond to the prevention of thousands of deaths (see Rosnow & Rosenthal, 1989).

This perspective is consistent with Rosenthal’s broader approach to effect sizes, which emphasizes the use of *r* as a common metric ( $r=Z/\sqrt{N}$ ) and advocates for its interpretation in more concrete terms. In particular, Rosenthal and Rubin’s (1982) binomial effect size display (BESD) translates *r* into differences in success rates, thereby illustrating how even numerically small effects can correspond to meaningful differences in outcomes (see other common language estimators in Metsämuuronen, 2025).

Nevertheless, although effect size benchmarks are context-dependent when interpreted in terms of practical importance, standardized classifications such as those proposed by Cohen (1988) and extended by Sawilowsky (2009) remain useful for methodological and comparative purposes. In particular, they provide a common metric for describing the relative magnitude of effects in standardized units, facilitate comparisons across studies and research domains, and enable the characterization of effect size distributions in meta-analytic work. In this sense, such benchmarks should not be understood as indicators of substantive importance, but rather as descriptive categories of statistical magnitude.

### Cohen’s d and r as Effect Sizes

There are many estimators of the ES in the population, developed for different purposes. Huberty (2002) and Peng & Chen (2014) have typologized these. Based on evaluations by Funder & Ozer (2016); Peng & Chen (2014); Schäfer & Schwarz (2019), two most frequently reported ES estimators are *d* and *r*. Their relationship is of specific interest because the traditional simplified thresholds for “small,” “medium,” or “large” effect sizes related to *r* are derived from those created for Cohen’s *d*. The thresholds for *d*, in turn, are based on percent overlap of two distributions which also seems a justified basis in the case of *R*_*PB*_. However, this rationale becomes unclear when comparing multiple populations, particularly if the number of cases in the subpopulations is radically imbalanced.

In the dichotomous case, $R_{PB}$ can be expressed on the scale of Cohen’s *d* using the traditional formula assuming subpopulations *i* and *j* with the corresponding number of cases *n*_1_ and *n*_2_:

(1) $$d_{1}=\frac{R_{PB}}{\sqrt{1-R_{PB}^{2}}}\left(\frac{n_{i}+n_{j}}{\sqrt{n_{i}\times n_{j}}}\right)=\frac{R_{PB}}{\sqrt{1-R_{PB}^{2}}}\times \frac{1}{\sqrt{p_{i}p_{j}}}=\frac{R_{PB}}{\sqrt{1-R_{PB}^{2}}}\times \frac{1}{\sqrt{p_{i}\left(1-p_{i}\right)}}$$

(derived from Cohen, 1988, p. 24), where *p*_i_ is the proportion of either of the subpopulations, and $p_{1}p_{2}=p_{1}\left(1-p_{1}\right)=p_{2}\left(1-p_{2}\right)$ . The *R*_*PB*_, in turn, is computed by using the traditional formula for PMC:

(2) $$R_{PB}=\rho_{gX}=\frac{\sigma_{gX}}{\sigma_{g}\sigma_{X}}=\left(\mu_{2}-\mu_{1}\right)\frac{\sqrt{p_{i}\left(1-p_{i}\right)}}{\sigma_{X}},$$

where *g* refers to a variable with two categories, *X* is a metric variable with ordinal, interval, or continuous scale, and *μ*_1_ and *μ*_2_ are the means of *X* in the subpopulation 1 and 2, respectively. Conversion of *d* to the scale of *r* is done using the inverse formula:

(3) $$R_{PB}=d/\sqrt{d^{2}+\frac{1}{p_{i}\left(1-p_{i}\right)}}$$

(Cohen, 1988, p. 24).

### Challenges in the Traditional Thresholds Related to the Magnitude of Correlation

Traditional thresholds for Cohen’s *d* are used as an overall benchmark and basis for the related estimators such as *r* and Cohen’s *f*. The traditional benchmarks for “small,” “medium,” and “large” *d* are given 0.2, 0.5, and 0.8, respectively (Cohen, 1969, 1988), extended by “very small,” “very large,” and “huge” *d* as 0.1, 1.2, and 2.0, respectively (Sawilowsky, 2009). Then, if we observe *R*_*PB*_ = 0.25, and the number of cases in the subpopulations was identical (*p*_*i*_ = 0.5), based on equation (1), the corresponding *d* would be $d_{1}=0.25/\sqrt{\left(1-0.25^{2}\right)\cdot \left(0.5\cdot 0.5\right)}=0.52$ , indicating a “medium” effect size. However, if most cases come from one subpopulation (e.g., 95 % of the cases) and the other subpopulation has few cases (consequently, 5%), the same correlation turns to be “very high” ( $d_{1}=0.25/\sqrt{\left(1-0.25^{2}\right)\cdot \left(0.95\cdot 0.05\right)}=1.18$ ).

This information does not appear to be widely used. This can be inferred from the continued presentation of simplified forms and consequent simplified thresholds presented in tutorial materials (e.g., Statistics How To, 2026; UCLA, 2026; Wikipedia, 2026; see however, e.g., Lenhard & Lenhard, 2022), in software packages (e.g., https://cran.r-project.org/web/packages/effectsize/vignettes/convert_r_d_OR.html), and textbooks (e.g., Borenstein et al., 2009). Even serious research articles allude to these simplified forms by using thresholds based on them (e.g., Correll et al., 2020; Gignac & Szoborai, 2016; Funder & Ozer, 2016; Kim, 2016; see, however, e.g., McGrath & Meyer, 2006).

Another challenge regarding *r* effect size is that traditional tables for transforming *r* to *d* and vice versa (Cohen, 1988, p. 22) assume equal sample sizes (p. 23). This is, obviously, a rare case in most applications. Nevertheless, based on this simplification, the thresholds for *r* in the point-biserial settings are traditionally been 0.1, 0.24, and 0.37 for “small,” “medium,” and “large” ES, respectively, and 0.1, 0.3, and 0.5 for polytomous and continuous cases. The latter are based on transforming the estimate for *R*_*PB*_ to the biserial correlation (*R*_*B*_) using another simplified formula *R*_*B*_ = *R*_*PB*_ × 1.253 (Cohen, 1988, p. 82). The latter thresholds appear *too high* when applied in the polytomous settings. This conclusion is supported by empirical findings from, for example, Gignac & Szoborai, 2016 and Funder and Ozer (2016) who propose that, instead of Cohen’s standards 0.1–0.3–0.5 for “small,” “medium,” and “large” ES, the thresholds should be closer to 0.1–0.2–0.3, respectively. Later in this article, it will be shown that the general formulae derived here closely support these latter thresholds.

## Research Problem and the Course of Study

It is unsatisfactory and potentially misleading to apply standards developed for dichotomous settings directly to polytomous and continuous settings. The challenge lies in the absence of transformation formulas that provide the same level of exactness as equation (1) in dichotomous settings.^1^

The following section derives generalized formulae enabling accurate transformation of correlation estimates from *R*_*PB*_ and continuous variables to the scale of *d* and vice versa. This approach provides comparable estimates of effect sizes in dichotomous, polytomous, and continuous settings. One advantage of this general form is that the verbal descriptors of effect sizes and numerical values of effect sizes correspond between Cohen’s *d* and *r*, regardless of discrepancies in subpopulation sizes or category counts.

In the main text, the number of formulas is kept to a minimum. The derivations are located in Appendix 1.

## General Formulae for Transforming *r* to the Scale of *d*

### General Formulae

Although no general formula for polytomous settings has been available, it is reasonable to consider equation (1) as a special case and reduced form of a more general formula for converting *r* to the scale of *d*.^2^ It is reasonable to assume that, instead of the term $\sqrt{p_{i}p_{j}}$ , the general form includes the term $\overset{R}{\underset{i\neq j}{\sum }}p_{i}p_{j}/R\left(R-1\right)$ , that is, the *average* of all $p_{i}p_{j}$ , where $p_{i}\neq p_{j}$ , $p_{i}p_{j}\neq p_{j}p_{i}$ , and R(R−1) is the number of all combinations of $p_{i}p_{j}$ . Using straightforward algebra (see Appendix 1), we can express the general form for transforming *r* to the scale of *d* as follows (equation (8) in Appendix 1):

(4) $$d_{8}=\frac{\rho_{gX}}{\sqrt{1-\rho_{gX}^{2}}}\times \sqrt{\frac{1}{\overset{R}{\underset{i\neq j}{\sum }}p_{i}p_{j}}}\times \sqrt{\frac{4\left(R-1\right)}{R}}.$$

Because $\overset{R}{\underset{i\neq j}{\sum }}p_{i}p_{j}$ = $\overset{R}{\underset{i=1}{\sum }}p_{i}\left(1-p_{i}\right)$ , we get an alternative form of the general formula as follows (equation (9) in Appendix 1):

(5) $$d_{9}=\frac{\rho_{gX}}{\sqrt{1-\rho_{gX}^{2}}}\times \sqrt{\frac{4\left(R-1\right)}{R\overset{R}{\underset{i=1}{\sum }}p_{i}\left(1-p_{i}\right)}}.$$

The opposite general transformation from *d* to the scale of *r*, parallel to that of equation (3), is as follows:

(6) $$\begin{array}{l} \rho_{gX}=d_{8}/\sqrt{d_{8}^{2}+\frac{4\left(R-1\right)}{R\overset{R}{\underset{i\neq j}{\sum }}p_{i}p_{j}}}\\ \;\;\;\;\;\;=d_{9}/\sqrt{d_{9}^{2}+\frac{4\left(R-1\right)}{R\overset{R}{\underset{i=1}{\sum }}p_{i}\left(1-p_{i}\right)}} \end{array}$$

The variance and confidence interval for the *d* estimate can be obtained partly from the traditional formulae. The approximate variance of the *r* is $V_{r}=\frac{1-r^{2}}{n-1}$ . In the general case involving *d*_8_ and *d*_9_, the variance of *d* (*V*_*d*_) is given by:

(7) $$V_{d_{8}}=\frac{\left(R-1\right)}{R\overset{R}{\underset{i\neq j}{\sum }}p_{i}p_{j}}\cdot \frac{4V_{r}}{{\left(1-r^{2}\right)}^{3}}=\frac{\left(R-1\right)}{R\overset{R}{\underset{i=1}{\sum }}p_{i}\left(1-p_{i}\right)}\cdot \frac{4V_{r}}{{\left(1-r^{2}\right)}^{3}}=V_{d_{9}}$$

(equation (12) in Appendix 1). Note that the transformations in equations (5) and (6) are derived for an ordinal categorical grouping variable paired with a continuous variable. However, the formulation generalizes naturally to the continuous–continuous setting. In the limiting case where the “grouping” variable is fully continuous—that is, when all values are unique—the category proportions become uniform (*p*_*i*_ = *p*_*j*_ for all *i*, *j*), yielding equal weights across observations. Under these conditions, equation (7) reduces to the traditional form $V_{d}=\frac{4V_{r}}{{\left(1-r^{2}\right)}^{3}}$ (e.g., Borenstein et al., 2009). The 95% confidence interval for *d*_9_ can be estimated by

(8) $$CI95\%=d\pm {t_{95\%,}}_{\left(n-2\right)}\sqrt{V_{d}}$$

### Special Forms of the General Formulae

In dichotomous case, where *R* = 2, the general formulae *d*_8_ and d_9_ are reduced to the traditional form:

(9) $$\left\{ \begin{array}{l} d_{8}=\frac{\rho_{gX}}{\sqrt{1-\rho_{gX}^{2}}}\times \sqrt{\frac{1}{p_{1}p_{2}+p_{2}p_{1}}}\times \sqrt{\frac{4\left(2-1\right)}{2}}=\frac{\rho_{gX}}{\sqrt{1-\rho_{gX}^{2}}}\times \frac{1}{\sqrt{p_{1}p_{2}}}=d_{1}\;\\ d_{9}=\frac{\rho_{gX}}{\sqrt{1-\rho_{gX}^{2}}}\times \sqrt{\frac{1}{p_{1}\left(1-p_{1}\right)+p_{2}\left(1-p_{2}\right)}}\times \sqrt{\frac{4\left(2-1\right)}{2}}=\frac{\rho_{gX}}{\sqrt{1-\rho_{gX}^{2}}}\times \frac{1}{\sqrt{p_{1}\left(1-p_{1}\right)}}=d_{1} \end{array}\right..$$

The general formulae (*d*_8_ and *d*_9_) also reduce to the traditional transformation under specific limiting conditions. This occurs, first, in the fully continuous case, where the “grouping” variable contains only unique values, and second, more generally, in ordinal categorical settings in which all subpopulations are equally sized (i.e., *p*_*i*_ = *p*_*j*_ for all *i*, *j*). The latter situation may arise, for example, when a polytomous grouping variable is constructed using equally sized categories (e.g., in experimental designs, quantiles, or balanced discretizations). Under these conditions, the weighting structure becomes uniform, and the proposed transformation collapses to the classical form^3^:

(10) $$d_{13}=\frac{\rho_{gX}}{\sqrt{1-\rho_{gX}^{2}}}\times 2=\frac{2\rho_{gX}}{\sqrt{1-\rho_{gX}^{2}}}$$

(see equation (13) in Appendix 1 and the related derivation). Importantly, no assumption of equal category proportions is required for the validity of the proposed transformation, which remains applicable for arbitrary values of *p*_*i*_. The condition *p*_*i*_ *= p*_*j*_ merely defines a special case under which the general formulation coincides with the classical Cohen transformation. This provides a theoretical justification for the empirical robustness of the classical r-to-d transformation in settings where the grouping variable is not strictly continuous but has a sufficiently large number of ordered categories. In the case of equal proportions, because of equation (6), equation (9) reduces to the form

(11) $$\rho_{gX}=d_{8}/\sqrt{d_{8}^{2}+4},$$

which corresponds to the traditional form of the simplified transformation formula (Cohen, 1988). Nevertheless, this robustness applies primarily when the second variable remains continuous. When both variables are ordinal or otherwise discretized, additional attenuation arises due to the loss of information in both margins. In such cases, the classical transformation may no longer provide an accurate approximation, and the error depends jointly on the number of categories and their marginal distributions.^4^

### Traditional Formula as a Shortcut

In some cases, detailed information about discrepancies between subpopulations may be unavailable in research reports. Then, in applied contexts, including meta-analysis, it is useful to have a reliable shortcut formula for the transformation.

One such shortcut is the traditional formula $d_{17}=2\rho_{gX}/\sqrt{1-\rho_{gX}^{2}}$ (Cohen, 1988, p. 23; equation (17) in Appendix 1), which assumes equally distributed subpopulations. When using the shortcut form *d*_17_, the accuracy of transforming *r* to the *d* scale depends on the proportions *pi* and *p*_*j*_. The greater the disparity between these proportions, the larger the deviation of the traditional shortcut from the true value. This phenomenon is illustrated below with two examples drawn from real-life settings.

### Numerical Examples of the Estimators in Real-Life Settings

Case 1: Shortcut estimator succeeds in reaching the true effect size

In this example, we examine the magnitude of the relationship between two independent measures of students’ achievement levels. The data are drawn from published results of a national assessment of mathematics learning outcomes in Finland (Metsämuuronen, 2023b). Teachers’ marks, expressed on an ordinal scale ranging from 5 (“passable”) to 10 (“excellent”), are compared with the students’ scores on the national test. The condensed results are shown in Table 1.

**Table 1.** Statistics for transforming r to the scale of d in a real-life setting with 6 subpopulations

Teachers’ marks	Mean at a national test	Std. deviation	*N*	pi	pi (1−p_i_)
5	306.0	78.216	459	0.0369	0.0355
6	343.6	68.037	1,984	0.1595	0.1341
7	389.0	73.980	2,570	0.2066	0.1639
8	452.6	73.854	2,929	0.2355	0.1800
9	523.2	72.031	2,886	0.2320	0.1782
10	599.9	76.910	1,610	0.1294	0.1127
Total	452.1	113.284	12,438	SUM =	0.8045
​	​	​	R_gX_ = 0.7582		​
​	​	​	Var(r) = 0.0000145		​
​	​	Equation (in Appendix 1)	Estimates	​	Effect size in a verbal form
​	*d*_*8*_ exact	8	2.3672	​	“Huge”
​	*d*_9_ exact	9	2.3672	​	“Huge”
​	*Var(d*_8_ *), Var(d*_9_ *)*	11	0.0007837	​	​
​	*CI95%*	14	[2.313, 2.421]	​	​
​	*d*_17_ shortcut	17	2.3258	​	“Huge”
​	*Var(d*_17_ *)*	12	0.000757	​	​
​	*CI95%*	14	[2.272, 2.380]	​	​

As expected, the variables show a strong correlation (*R*_*gX*_ = 0.758). The difference between the extreme proportions of subpopulations is modest, *p*_*d*_ = 0.2355–0.0369 = 0.1986, while the overall distribution is skewed-normal. Given this relatively low discrepancy index *p*_*d*_, indicating that *p*_*i*_ ≈ *p*_*j*_, the shortcut estimator is expected to approximate the true effect size closely.

The figures necessary for computing the transformations are collected in Table 1. The estimates *d*_8_ and *d*_9_ are computed as follows:

*d*_8_ = *d*_9_ = $\frac{0.7582}{\sqrt{1-0.7582^{2}}}\times \sqrt{\frac{1}{0.8045}}\times \sqrt{\frac{4\times 5}{6}}=2.3672$ .

The estimate by the traditional shortcut estimator is as follows:

*d*_17_ = $\frac{2\cdot 0.7582}{\sqrt{1-0.7582^{2}}}$ = 2.3258.

We observe that the estimate by *d*_17_ is very close to the exact value, and the interpretation is the same: the correlation is classified as “huge.”

For the confidence interval, the traditional variance of *r* is computed as follows: $V_{r}=\frac{1-r^{2}}{n-1}=$ $\frac{1-0.7582^{2}}{12,438-1}$ = 0.0000145, and the variance of *d*_8_ and *d*_9_ is computed as follows by equation (7): $V_{d_{8}}=V_{d_{9}}=\frac{4V_{r}\cdot \left(R-1\right)}{\left({\left(1-r^{2}\right)}^{3}\cdot R\overset{R}{\underset{i=1}{\sum }}p_{i}\left(1-p_{i}\right)\right)}=$ $\frac{4\cdot 0.0000145\cdot 5}{{\left(1-0.7582^{2}\right)}^{3}\cdot 6\cdot 0.8045}$ = 0.000784. Then, the 95% confidence interval for *d*_8_ and *d*_9_ is $CI95\%=2.3672\pm 1.96\sqrt{0.000784}=$ [2.313, 2.421]. For *d*_17_, the traditional variance of d can be used: $V_{d17}=\frac{4V_{r}}{{\left(1-r^{2}\right)}^{3}}=$ $\frac{4\cdot 0.0000145}{{\left(1-0.7582^{2}\right)}^{3}}$ = 0.000757, and the corresponding 95% confidence interval is $CI95\%=2.3258\pm 1.96\sqrt{0.000757}$ = [2.272, 2.380]. Note that the variance of *r* is negatively biased with high values of point-biserial and point-polyserial correlation values because these estimators always produce deflated estimates with high correlations (see, e.g., Metsämuuronen et al., 2025).

Case 2: Shortcut estimator fails radically.

In the second example, students’ achievement levels are categorized into three groups based on the intensity of support needed for their studies. The data, originally reported by Metsämuuronen, (2023b) and also analyzed in terms of Cohen’s *f* by Metsämuuronen (2026), are here reanalyzed using estimators related to *r* and *d*.

In the dataset, 86% of the students received general support, 10% intensified support, and 4% special support. The discrepancy index, *p*_*d*_ = 0.8587−0.0408 = 0.8179, indicates a pronounced imbalance between subpopulation sizes (Table 2). Consequently, the shortcut estimator is expected to considerably underestimate the true effect size.

**Table 2.** Statistics for transforming r to the scale of Cohen’s d in a real-life setting

Subpopulation	Group	Mean	Std. deviation	*N*	pi	p_i_ (1–p_i_)
0	General support	468.7	107.3593	10,493	0.8587	0.1213
1	Intensified support	358.9	91.7799	1,228	0.1005	0.0904
2	Special support	332.9	98.4396	498	0.0408	0.0391
​	Total	452.2	113.2576	12,219	SUM =	0.2508
​	​	RgX = −0.3512		​	​	​
​	​	Var(r) = 0.0000629		​	​	​
​	Equation (in Appendix 1)		Cohen d	Effect size in a verbal form		​
*d*_8_ exact	8		−1.2231	“Very large” or “very high”		​
*d*_9_ exact	9		−1.2231	“Very large” or “very high”		​
*Var(d*_8_*)*, *Var(d*_9_*)*	11		0.00099	​		​
*CI95%*	14		[−1.285, −1.161]	​		​
*d*_17_ shortcut	17		−0.7502	“large” or “high”		​
*Var(d*_17_ *)*	12		0.00037	​		​
*CI95%*	14		[−0.788, −0.712]	​		​

The categorical variable is ordinal, representing the amount of support required: no specific support (0), intensified support (1), and special support (2). Consequently, the point-polyserial correlation is appropriate. As higher achievement corresponds to less support, the correlation is negative (*R*_*gX*_ = −0.3512).

The computations are straightforward. The estimates by *d*_8_ and *d*_9_ are

$$d_{8}=d_{9}=\frac{-0.3512}{\sqrt{1-0.3512^{2}}}\times \sqrt{\frac{1}{0.2508}}\times \sqrt{\frac{4\times 2}{3}}=-1.2231$$

while the shortcut estimator gives the following:

$$\frac{2\cdot \left(-0.3512\right)}{\sqrt{1-0.3512^{2}}}=-0.7502$$

The other statistics are calculated the same way as with Table 1. They are not repeated here.

Clearly, the shortcut estimator substantially underestimates the effect size. When the discrepancy in subpopulation proportions is accounted for in the transformation, a “high” correlation with a “large” effect size (ǀ*d*ǀ = 0.75) becomes a “very high” correlation with a “very large” effect size (ǀ*d*ǀ = 1.22).

The difference in the transformation results for case 2 can be evaluated by using the *PHD* (probability of higher group dominance based on Somers’ delta) estimator, a common-language estimator of ES (Metsämuuronen, 2025). The result *d* = −0.75 transforms to *PHD* ≈ 0.32 while *d* = −1.22 transforms to *PHD* ≈ 0.25 (see Metsämuuronen, 2024). A *PHD* value of 0.32 means that, if 100 pairs of students are randomly selected from the population, 32% of the pairs are expected to consist of a student who needs more academic support in studies but performs better on the mathematics test. In reality, a closer approximation would be only 25% of such pairs (*PHD* = 0.25). This seven-percentage-point difference illustrates the underestimation by the shortcut estimator, which is consistent with its known behavior under conditions of extreme subpopulation imbalance.

These two examples illustrate that the traditional shortcut formula for converting *r* to the scale of *d* can yield accurate results when subpopulation proportions are close each other, as shown in Case 1. However, it substantially underestimates the effect size when the proportions differ greatly, as shown in Case 2.

### Refined Thresholds for “Small,” “Medium,” and “Large” r Effect Size

What is considered “large” or “high” in one context may be considered “small” in another. For example, in item analysis, *R*_*gX*_ = 0.24 is considered a “low” item-total correlation even though, according to Cohen’s standards, it is considered “medium.” Thus, verbalizing the practical meaning of the effect sizes is ambiguous. Here, however, we discuss the traditional thresholds.

In the binary and dichotomous settings, the traditional thresholds of *r* effect size thresholds are 0.05, 0.1, 0.24, 0.37, 0.51, and 0.71 for “very small,” “small,” “medium,” “large,” “very large,” and “huge” ES, respectively (Cohen, 1988; Sawilowsky, 2009). These thresholds are accurate when subpopulations are *equal in size* and when variables are *continuous*, which is a special case of the condition *p*_*i*_ = *p*_*j*_ (see equation (6)). However, in many practical settings where point-biserial and point-polyserial correlations are used, these traditional thresholds are inaccurate or even misleading because the condition of *p*_*i*_ = *p*_*j*_ is a special case amidst the designs used in experimental and quasi-experimental studies. Equations (4) and (5) provide access to accurate transformations for dichotomous, polytomous, and continuous cases also including the special case of *p*_*i*_ = *p*_*j*_.

Discrepancies in subpopulation sizes affect the transformation results. The statistic called the discrepancy index, *p*_*d*_ = *p*_*max*_ − *p*_*min*_, where *p*_*d*_ = 0 corresponds to equal subpopulation sizes, can be used as a rough guide to assess the accuracy of transforming *R*_*gX*_ to the scale of Cohen’s *d*. When *p*_*d*_ ranges from 0 to 0.4, the widened threshold ranges are 0.05 for “very small,” 0.09–0.10 for “small,” 0.22–0.24 for “medium,” 0.34–0.37 for “large” or “high,” 0.48–0.51 for “very large” or “very high,” and 0.68–0.71 for “huge” correlation. The range indicates that a correlation of 0.68, for example, should be interpreted as “huge” if the discrepancy between the largest and smallest subpopulation proportions is 0.4 while the corresponding threshold is 0.71 with equal subpopulation sizes. This level of accuracy (0.68–0.71) may be sufficient for many practical settings where evaluations of correlation magnitude are needed. The corresponding widened boundaries for *d* are the following: 0.10–0.11 for “very small,” 0.20–0.22 for “small,” 0.50–0.55 for “medium,” 0.80–0.87 for “large,” 1.20–1.31 for “very large,” and 2.00–2.18 for “huge” effect size. The lower boundary refers to *p*_*d*_ = 0.0 and the upper boundary to *p*_*d*_ = 0.4.

**Figure 1.**
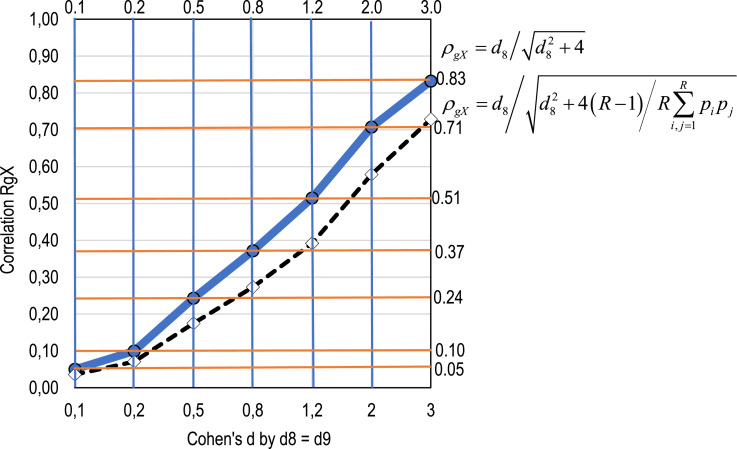
Relation of r and d assuming that p_i_ = p_j_ (blue solid line) and exact correspondence when R = 3, p_1_ = 0.80, p_2_ = 0.15, and p_3_ = 0.05, p_d_ = 0.75 (black dashed line)

The effect of *p*_*d*_ is illustrated in Figure 1 as a wide, inaccurate line.

In conclusion, when *p*_*d*_ < 0.40, the traditional shortcut estimator assuming equal subpopulation proportions could be used in the transformation process. For larger discrepancies (*p*_*d*_ > 0.4), however, it is recommended to use the general estimator *d*_8_ or *d*_9_. In all cases, except the case *p*_*i*_ = *p*_*j*_, the general estimators give more precise estimate.

## Discussion, Suggestions, and Restrictions

### Main Results in a Nutshell

This article originated from the observation that Cohen’s *d* has traditionally served as the benchmark for the magnitude of the *r* effect size. While an accurate transformation between *r* and *d* exists for the point-biserial correlation, no such a formula has been available for polytomous settings. To address this gap, two general formulae covering dichotomous, polytomous, and continuous cases were derived to provide a robust transformation.

The main results can be condensed as follows.

(1) The general formulae *d*_8_ and *d*_9_ yield an exact correspondence between *r* and *d,* irrespective of the number of categories in *g*, or the discrepancy in subpopulation sizes.
(2) The traditional formula *d*_17_, which assumes equal subpopulation sizes, provides a close approximation of the true correspondence when subpopulation sizes are close to each other.
(3) When the discrepancy is moderate (i.e., *p*_*d*_ < 0.40), the refined qualitative thresholds are 0.05 for “very small,” 0.09–0.10 for “small,” 0.22–0.24 for “medium,” 0.34–0.37 for “large,” 0.48–0.51 for “very large,” and 0.68–0.71 for “huge” correlations. These thresholds are notably lower than the traditional values based on Cohen’s (1988) suggestions for biserial correlation, which have sometimes been applied to polytomous and continuous settings as well. Nevertheless, the refined boundaries correspond closely with empirical findings reported by Funder & Ozer (2016)and Gignac & Szoborai (2016).
(4) When the discrepancy is very high (*p*_*d*_ > 0.4)—that is, when one subpopulation accounts for a majority of cases (e.g., 70–80% or more), and the others are much smaller (e.g., 10% or less)—the traditional shortcut estimator may severely underestimate the effect size. The general formulae yield an exact correspondence between *r* and *d* even under large or extreme discrepancies.

### Suggestions for Applied Users

To summarize the recommended procedure for transforming Pearson’s *r* to the scale of Cohen’s *d* and assigning qualitative labels such as “small,” “medium,” or “large,” the following steps should be taken.

If your correlation comes from two *continuous variables* or if the *group sizes are equal* (i.e., *p*_*i*_ = *p*_*j*_) or nearly equal (*p*_*i*_ ≈ *p*_*j*_), you can use the following procedure.

(1) Calculate the estimate of *R*_*XY*_ between the two continuous variables.
(2) Use the traditional thresholds for *r* strictly: 0.05 for “very small,” 0.10 for “small,” 0.24 for “medium,” 0.37 for “large,” 0.51 for “very large,” and 0.71 for “huge” correlation. Note that the verbal annotations depend on the context: what is “small” in one context may be “large” in another.
(3) Alternatively, use the transformation formula $d_{17}=2\rho_{XY}/\sqrt{1-\rho_{XY}^{2}}$ to transform *r* to the *d* scale and use the corresponding thresholds given for Cohen’s *d*: 0.1 for “very small,” 0.2 for “small,” 0.5 for “medium,” 0.8 for “large,” 1.2 for “very large,” and 2 for “huge” effect size.

If your variables are *not continuous*, the following procedure can be used to transform *r* to a *d* scale.

(1) Calculate the estimate of *R*_*gX*_ between variables *g* and *X*, where *g* refers to a variable with a narrower scale, either dichotomous or ordinal, and *X* is a metric variable with an ordinal, interval, semi-continuous, or continuous scale. Let the number of categories in *g* be *R.*
(2) Calculate the proportion of cases in each group of variable *g* (*p*_*i*_). *R* and *p*_*i*_ are needed to transform *r* to the *d* scale.
(3) Use the following formula to transform the estimates of *r* to the *d* scale: $d_{9}=\frac{\rho_{gX}}{\sqrt{1-\rho_{gX}^{2}}}\times \sqrt{\frac{4\left(R-1\right)}{R\overset{R}{\underset{i=1}{\sum }}p_{i}\left(1-p_{i}\right)}}$ .

As an aid, Appendix 2 includes an R script for transforming Pearson’s *r* to the scale of Cohen’s *d* from the given *R*_*gX*_ (point-biserial and point-polyserial case) or *R*_*XY*_ (continuous case). An Excel file is also provided for non-R users. Note that the approximation is better when the other variable is continuous, and it gets less accurate when the scale of the variable with the wider scale gets narrower.

(4) When the *r* estimates are transformed to the scale of *d*, use the standard benchmarks developed for Cohen’s *d*: approximately 0.1 for “very small,” 0.2 for “small,” 0.5 for “medium,” 0.8 for “large,” 1.2 for “very large,” and 2 for “huge” effect size.

If your correlation comes from two *non-continuous variables* with a *small imbalance* in subpopulation sizes (*p*_*d*_ < 0.40), you can use the following simplified procedure.

(1) Calculate the estimate of *R*_*gX*_ between variables *g* and *X*, where *g* refers to the variable with a narrower scale with dichotomous or ordinal scale and *X* is metric variable with on ordinal, interval, semi-continuous, or continuous scale.
(2) Use the widened thresholds strictly: 0.05 for “very small,” 0.09–0.10 for “small,” 0.22–0.24 for “medium,” 0.34–0.37 for “large,” 0.48–0.51 for “very large,” and 0.68–0.71 for “huge” correlation. The lower boundary refers to *p*_*d*_ = 0.40 and the upper boundary to *p*_*d*_ = 0.0.
(3) Alternatively, use the transformation formula $d_{17}=2\rho_{gX}/\sqrt{1-\rho_{gX}^{2}}$ and the corresponding widened boundaries for *d*: 0.10–0.11 for “very small,” 0.20–0.22 for “small,” 0.50–0.55 for “medium,” 0.80–0.87 for “large,” 1.20–1.31 for “very large,” and 2.00–2.18 for “huge” effect size. The lower boundary refers to *p*_*d*_ = 0.0 and the upper boundary to *p*_*d*_ = 0.4.
(4) Note that when the largest discrepancy between subpopulation proportions exceeds 0.40, the large absolute values of *r* are substantially underestimated, and the traditional formula may substantially underestimate *d.*

### Known Restrictions

Although the new transformation formulae for *r*-estimates are general in the sense that they are not restricted to any specific scale in the variables, the estimators are still based on “justified reasoning.” The fact that the transformation formulae *d*_8_ and *d*_9_ produce the same estimates as the traditional estimators (*d*_1_ and *d*_17_) in special cases does not mean that they are “correct.” However, we may note that (1) the basis of the formulae is justified, (2) the reduced forms fit the theory in the binary and dichotomous settings as well as (3) they fit in cases with an equal number of cases in the categories including continuous cases, (4) shortcut benchmarking estimators give largely similar outcome, (5) the outcome makes sense, and (6) it fits well with the empirical findings of independent researchers regarding the refined thresholds for different levels of effect sizes. Nevertheless, systematic studies with the estimators would be beneficial.

Switching from a traditional *d* based on two subpopulation distributions to a generalized *d*, which is not limited to two distributions, may obscure the visual interpretation of *d* based on the overlap of these populations. This is particularly true in the continuous case, where there are no subpopulation distributions at all. This issue was not discussed in the text. It requires its own treatment. Nevertheless, since Cohen generalized the *r* scale thresholds to the continuous biserial correlation scale, and since many *r* estimators are transformed to *d* in any case, this issue does not seem insurmountable.

## Supplemental Material

Supplemental Material - General formulae for transforming Pearson’s r to the scale of Cohen’s d Supplemental Material for General formulae for transforming Pearson’s r to the scale of Cohen’s d by Jari Metsämuuronen in Applied Psychological Measurement.

Supplemental Material - General formulae for transforming Pearson’s r to the scale of Cohen’s d Supplemental Material for General formulae for transforming Pearson’s r to the scale of Cohen’s d by Jari Metsämuuronen in Applied Psychological Measurement.

## Data Availability

No specific dataset is used in the empirical section. The examples are based on published results.*

## Notes

1. Within the text, the term “exact” refers to transformations that explicitly incorporate the number of categories and their distributional imbalance, in contrast to commonly used shortcut estimators that ignore these features. In this sense, “exact” denotes a fully specified transformation within the proposed framework rather than an assumption-free or sample-level notion of exactness. It is worth noting that an exact relationship between *r* and *d* already exists in the classical dichotomous case (*R* = 2; Cohen, 1988), where the transformation is an algebraic identity. However, this exact formulation is not consistently applied in practice, and simplified approximations are often used instead. The present approach generalizes this exact relationship to ordinal settings by explicitly accounting for category structure, while preserving the classical result as a special case. Thus, “exact” should be understood as referring to a fully specified, design-consistent transformation that recovers the classical identity when *R* = 2 and provides a more accurate alternative to approximate conversions in more general settings.

2. This approach leads to the concept of “generalized *d,*” which is proposed by Metsämuuronen (2026). In this case, the *d* effect size refers to the overlap of several populations, rather than just two. In generalized *d*, *R* is the number of distributions to compare. This generalizes to continuous cases too, even though the distribution overlap is irrelevant in those cases.

3. Note that Rosenthal (1994) presents this form as a *general* transformation from *r* to *d*. However, it is important to recognize that this formula implicitly assumes that the observed correlation adequately reflects the underlying association, and is typically derived under assumptions of normality and continuity of the variables. In settings involving discretized or highly imbalanced variables, these assumptions do not generally hold. It is well known that correlation coefficients are attenuated under discretization and tend to approach zero in the presence of extreme marginal imbalance, even when the underlying relationship is strong or even perfect. In such cases, the mapping between *r* and *d* is not invariant but depends on the measurement structure, including the number of categories and their distribution. The generalized formulae proposed here address this limitation by explicitly modeling how the r-to-d transformation depends on category structure and imbalance. In this sense, the proposed formulation complements algebraic transformations such as those of Cohen (1988) and Rosenthal (1994) by making explicit the conditions under which such mappings are accurate or biased.

4. An anonymous reader raised an important point regarding the possible challenges in interpretation of *r* in scale of *d* when *r* is usually considered reversible but *d* is not. Although Pearson’s r is algebraically symmetric, this symmetry does not generally extend to the measurement level when variables differ in scale or level of resolution (see Metsämuuronen, 2022, 2023a, 2023b). In particular, when one variable is continuous and the other is binary or ordinal, the relationship is effectively directional: the higher-resolution variable can be seen as explaining variation in the lower-resolution variable, whereas the reverse interpretation entails a loss of information. This asymmetry is reflected in measurement modeling contexts, where aggregate scores are used to explain item-level responses rather than vice versa. Metsämuuronen (2022) notes that the directional effect is remarkable when the scale of “continuous” variable is four times wider than the scale of the “grouping” variable.
