# Supplementary material for: General formulae for transforming Pearson’s r to the scale of Cohen’s d
Source: Appl Psychol Meas. 2026 Jul 2:01466216261465015. Online ahead of print. doi: 10.1177/01466216261465015 (PMC13328108; doi:10.1177/01466216261465015)
Supplement: Supplemental Material - General formulae for transforming Pearson’s r to the scale of Cohen’s d [file sj-pdf-1-apm-10.1177_01466216261465015.pdf]

## Appendix 1. General formulae for transforming r to the scale of d

Cohen's *d* is based on the common agreement that the "*t-test statistic without n*" is a valuable statistic for measuring the difference between two means. Similarly, the "generalized *d* based on Pearson's *η*" ([Author2]) is based on the suggestion that the "*F-test statistic without n*" would be a valuable statistic to measure the difference between *several* means. In both cases, a solid mathematical "proof" for either approach may be difficult if not impossible to derive. Nevertheless, assuming that we have agreed the original relation of Cohen's *d* and *f* and Pearson's *r*, we continue further in their relationship in the following derivations. The formulas are numbered according to the main text. Formulae 1–3 are discussed in the main text, and formulae 4 and beyond continue from there.

### *1. General formulae for transforming r to the scale of d*

Assume an ordinal- or interval-scaled variable *g* with the number of categories *R* and the metric variable *X* with the number of categories *C*, and $R \leq C$. The product-moment correlation between *g* and *X* ( $\rho_{gX}$ ) is of interest. From Eq. (1) it is known that, in the dichotomous settings,

$$d_4 = \frac{\rho_{gX}}{\sqrt{1-\rho_{gX}^2}} \times \frac{1}{\sqrt{p_i p_j}} = \frac{\rho_{gX}}{\sqrt{1-\rho_{gX}^2}} \times \frac{1}{\sqrt{p_i(1-p_i)}} \tag{4}$$

It is justified to reason that, instead of the term $\sqrt{p_i p_j}$ , the general form has a term

$$\frac{\sum_{i,j=1}^{R} p_i p_j}{R(R-1)}, \tag{5}$$

i.e., the average of all $p_i p_j$, where $p_i p_j \neq p_j p_i$ and $R(R-1)$ is the number of these elements. Then, an initial form of the general formula would be as follows:

$$d_6 = \frac{\rho_{gX}}{\sqrt{1-\rho_{gX}^2}} \times \sqrt{\frac{R(R-1)}{\sum_{\substack{i,j=1 \\ i \neq j}}^{R} p_i p_j} \times C}, \tag{6}$$

where the element *C* is reserved for the adjustments needed to the formula. Because the reduced form in the case of $R = 2$ is known (Eq. 4), it is easy to conclude with light algebra that, assuming that Eq. (5) is justified, the element *C* is the following:

$$C = \frac{2}{R}. \tag{7}$$

This gives us a general form to transform *r* to the scale of *d*:

$$d_8 = \frac{\rho_{gX}}{\sqrt{1-\rho_{gX}^2}} \times \sqrt{\frac{1}{\sum_{\substack{i,j=1\\ i\neq j}}^{R} p_i p_j}} \times \sqrt{\frac{4(R-1)}{R}} \\ = d_4 \cdot \sqrt{\frac{4(R-1)}{R}} \quad . \tag{8}$$

Because $\sum_{\substack{i,j=1\\ i\neq j}}^{R} p_i p_j = \sum_{i=1}^{R} p_i (1-p_i)$, we get an alternative form of the general formula as follows:

$$d_9 = \frac{\rho_{gX}}{\sqrt{1-\rho_{gX}^2}} \times \sqrt{\frac{1}{\sum_{i=1}^{R} p_i (1-p_i)}} \times \sqrt{\frac{4(R-1)}{R}} \,. \tag{9}$$

*2. Variance and confidence interval of $d_8$ and $d_9$*

The variance and confidence interval for the *d* estimate can be obtained partly using the traditional formulae. For the variance of *d*, the variance of *r* is needed. Traditionally, the variance of the *r* is $V_r = \frac{(1-r^2)^2}{n-1}$ (e.g., Borenstein et al. 2009). This assumes that variables are bivariate normal random variables and *n* is large. For small sample sizes, somewhat more accurate approximation is based on Fisher's z approximation: $V_{r_z} = \frac{(1-r^2)^2}{n-1}$. Neither of these are accurate with point-biserial and point-polyserial case when correlation is high. However, better approximations are not available without bootstrapping.

Let us denote $d_8 = d_9 = K \cdot \frac{\rho_{gX}}{\sqrt{1-\rho_{gX}^2}}$, where $K = \sqrt{\frac{4(R-1)}{R\sum_{\substack{i,j=1\\ i\neq j}}^{R} p_i p_j}} = \sqrt{\frac{4(R-1)}{R\sum_{i=1}^{R} p_i (1-p_i)}}$. The derivative of $d_8$ and $d_9$ with respect to $\rho$ is:

$$\frac{\partial d_9}{\partial \rho} = K \cdot \frac{\partial}{\partial \rho}\left(\frac{\rho}{\sqrt{1-\rho^2}}\right) = \left(\frac{K}{(1-r^2)^{3/2}}\right). \tag{10}$$

Using the delta method (see Ver Hoef, 2012), the variance of $d_8$ and $d_9$ is:

$$
\begin{cases}
V_{d_8} = \left( \dfrac{K}{\left(1-\rho^2\right)^{3/2}} \right)^2 Var(\rho) = \dfrac{K^2 \cdot Var(\rho)}{\left(1-\rho^2\right)^3} \\
\quad = \dfrac{(R-1)}{R\sum\limits_{\substack{i=1 \\ i\neq j}}^{R} p_i p_j} \cdot \dfrac{4V_r}{\left(1-r^2\right)^3} \\
V_{d_9} = \left( \dfrac{K}{\left(1-\rho^2\right)^{3/2}} \right)^2 Var(\rho) = \dfrac{K^2 \cdot Var(\rho)}{\left(1-\rho^2\right)^3} \\
\quad = \dfrac{(R-1)}{R\sum\limits_{i=1}^{R} p_i (1-p_i)} \cdot \dfrac{4V_r}{\left(1-r^2\right)^3}
\end{cases}
\tag{11}
$$

In the continuous case and in the dichotomous and polytomous cases with $p_i = p_j$, Eq. (11) reduces to

$$
V_d = \frac{4V_r}{\left(1-r^2\right)^3}, \tag{12}
$$

which is the traditional form of the variance of $d$ in respect to correlation (e.g., Borenstein et al. 2009). Consequently, 95% confidence interval for $d = d_8 = d_9$ can be estimated by

$$
CI95\% = d \pm t_{95\%,(n-1)} \sqrt{V_{d_8}} = d \pm t_{95\%,(n-1)} \sqrt{V_{d_9}} . \tag{13}
$$

Because $R_{gX}$ is deflated, i.e., too low when $r$ is high (see, e.g., [Author5]), the $V_r$ and, consequently, the confidence interval are inaccurate with high $r$. The same challenge applies to traditional estimators.

*3. Special cases of the general formulae*

In the case of two subpopulations, $R = 2$, and Eqs. (8) and (9) are reduced as follows:

$$
\begin{aligned}
d_8 &= \frac{\rho_{gX}}{\sqrt{1-\rho_{gX}^2}} \times \frac{1}{\sqrt{p_i p_j + p_j p_i}} \times \sqrt{\frac{4\times(2-1)}{2}} \\
&= \frac{\rho_{gX}}{\sqrt{1-\rho_{gX}^2}} \times \frac{1}{\sqrt{p_i (1-p_i)}} = d_9 = d_1
\end{aligned} . \tag{14}
$$

When the number of cases in the subpopulations are identical, that is, when $p_i = p_j$ regardless of $R$, then $p_i = 1/R$. Then,

$$
\sum_{\substack{i,j=1 \\ i\neq j}}^{R} p_i p_j = \sum_{i=1}^{R} p_i (1-p_i) = \sum_{i=1}^{R} \left( p_i - p_i^2 \right) = \left( 1 - R p_i^2 \right) = 1 - \frac{1}{R} = \frac{R-1}{R} . \tag{15}
$$

Because of (15),

$$\sqrt{\frac{4(R-1)}{R\sum_{\substack{i,j=1\\i\neq j}}^{R} p_i p_j}} = \sqrt{\frac{4(R-1)}{R\sum_{i=1}^{R} p_i(1-p_i)}} = \sqrt{\frac{4(R-1)}{R}\times\frac{R}{(R-1)}} = 2. \tag{16}$$

Consequently, because of (8), (9) and (16), assuming $p_i = p_j$, including the continuous case,

$$d_{17} = \frac{\rho_{gX}}{\sqrt{1-\rho_{gX}^2}}\times 2 = \frac{2\rho_{gX}}{\sqrt{1-\rho_{gX}^2}} \tag{17}$$

of which form is familiar from Cohen (1988, p. 23) for the transformation for $R_{PB}$ in the case that the proportions are equal. This form gives the exact transformation for the continuous cases, but may give radical underestimation if the proportions of subpopulations vary notably. However, it could be used as a shortcut when the imbalance in the number of cases in the subpopulations is not wide.

## *4. General formulae for transforming d to the scale of r*

The opposite transformation form of $d$ to the scale of $r$, parallel to that of Eq. (3), is as follows

$$\rho_{gX} = d_8 \Bigg/ \sqrt{d_8^2 + \frac{4(R-1)}{R\sum_{i=1}^{R} p_i(1-p_i)}} = d_9 \Bigg/ \sqrt{d_9^2 + \frac{4(R-1)}{R\sum_{\substack{i,j=1\\i\neq j}}^{R} p_i p_j}} \tag{18}$$

In the case of equal proportions, because of (12), Eq. (14) is reduced to the form

$$\rho_{gX} = d_8 \Big/ \sqrt{d_8^2 + 4}\,, \tag{19}$$

which is the traditional form of the transformation formula.

## *5. Evaluation of the general formulae*

Although the transformation formulae $d_8$ and $d_9$ for $r$-estimates are general in the sense that they are not restricted to any specific scale in the variables, the estimators are still based on "justified reasoning". The fact that the transformation formulae $d_8$ and $d_9$ produce the same estimates as the traditional estimators ($d_1$ and $d_{17}$) in special cases does not mean that they are "correct". However, we may note that

(1) the basis of the formulae is justified,

(2) the reduced forms fit the theory in the binary and dichotomous settings,

(3) they fit in cases with an equal number of cases in the categories including continuous cases,

(4) their shortcut benchmarking estimators give similar outcome under the same assumptions,

(5) the outcome makes sense, and

(6) the outcomes fit well with the empirical findings of independent researchers regarding the refined thresholds for different levels of effect sizes.

**References**


Borenstein, M., Hedges, L. V., Higgins, J. P. T., & Rothstein, H. R. (2009). *Introduction to Meta-Analysis*. Wiley. https://doi.org/10.1002/9780470743386

Ver Hoef, J. M. (2012). Who invented the delta method? *The American Statistician*, *66*(2), 124–127. https://doi.org/10.1080/00031305.2012.687494

## Appendix 2. R script for transforming *r* to the scale of *d* for given $R_{gX}$ and $p_i$, $p_j$

```
# This R script transforms effect size r to the scale of Cohen's d.
# It calculates d8 and d9 (generalized form) and d17 (traditional shortcut
# form)from given RgX and p_i in dichotomous and polytomous  settings, or
# Cohen's d ((d17) from RXY in continuous settings. The script functions so
# that the non-continuous case is the default. If the continuous case was
# used, the non-continuous case should be deactivated. The example is from
# Table 2.
# Since the variance of r, used in estimating the variance of d, is
# inaccurate for large values for r, a bootstrap option for the confidence
# intervals is also provided.

# Parameters
rm(list = ls())            # clears the memory if several estimates are given
N <- 12219                 # Sample size for variance calculation
n_boot <- 5000             # Number of bootstrap resamples

# If your variables are CONTINUOUS, use this
RXY <- NA                  # Add Pearson correlation between two continuous
variables X and Y

# If g is DICHOTOMOUS or POLYTOMOUS and X is a metric variable, use this
RgX <- -0.3512                     # Pearson correlation (rho_{gX});
p <- c(0.8587, 0.1005, 0.0408)   # Vector of group proportions p_i and p_j
(they should sum to 1)

# Load progress package for bootstrapping
if (!requireNamespace("progress", quietly = TRUE))
install.packages("progress")
library(progress)

# Function to compute d values with 95% CI and bootstrap
compute_d_values <- function(rho_gX = NULL, p = NULL, continuous_case =
FALSE, RXY = NULL, N = NULL, n_boot = 1000) {

  # Helper function for magnitude labels; CHANGE when needed less
conservative
  label_magnitude <- function(d) {
    abs_d <- abs(d)
    if (abs_d >= 1.951) return('"huge"')
    if (abs_d >= 1.151) return('"very large" or "very high"')
    if (abs_d >= 0.751) return('"large" or "high"')
    if (abs_d >= 0.4) return('"medium"')
    if (abs_d >= 0.151) return('"small"')
    if (abs_d >= 0.1) return('"very small"')
    return('"trivially small"')
  }

  # Determine which case to use
  continuous_case <- FALSE
  discrete_case <- FALSE

  if (!is.null(RXY) && !is.na(RXY) && is.numeric(RXY)) {
    continuous_case <- TRUE
    rho_gX <- RXY
  } else if (!is.null(rho_gX) && is.numeric(rho_gX) && !is.null(p) &&
all(!is.na(p))) {
```

```
    discrete_case <- TRUE
  }

  if (!continuous_case && !discrete_case) {
    stop("Provide either RXY for the continuous case or rho_gX and p for the
discrete case.")
  }

  # --- Continuous case ---
  if (continuous_case) {
    d17 <- 2 * rho_gX / sqrt(1 - rho_gX^2)

    # Traditional CI
    var_r <- (1 - rho_gX^2)^2 / (N - 1)
    se_d <- 2 / (1 - rho_gX^2)^(3/2) * sqrt(var_r)
    t_val <- qt(0.975, df = N - 2)
    ci_lower <- d17 - t_val * se_d
    ci_upper <- d17 + t_val * se_d

    # Output
    cat("d17: ", round(d17,4), " (", label_magnitude(d17), ")\n", sep = "")
    cat(" traditional CI95% [", round(ci_lower,4), ", ", round(ci_upper,4),
"]\n", sep = "")
    cat(" bootstrap CI95% not applicable (deterministic)\n")

    return(invisible(list(d17 = d17, ci_trad = c(ci_lower, ci_upper))))
  }

  # --- Discrete case ---
  if (discrete_case) {
    R <- length(p)
    sum_p_term <- sum(p * (1 - p))

    # Compute d values
    d9 <- rho_gX / sqrt(1 - rho_gX^2) * sqrt(1 / sum_p_term) * sqrt(4 * (R -
1) / R)
    d17 <- 2 * rho_gX / sqrt(1 - rho_gX^2)

    # Traditional CI for each d separately
    var_r <- (1 - rho_gX^2)^2 / (N - 1)
    se_d9 <- sqrt((1 / sum_p_term) * (4 * (R - 1) / R)) * 1 / (1 -
rho_gX^2)^(3/2) * sqrt(var_r)
    se_d17 <- 2 / (1 - rho_gX^2)^(3/2) * sqrt(var_r)
    t_val <- qt(0.975, df = N - 2)
    ci_d9 <- c(d9 - t_val * se_d9, d9 + t_val * se_d9)
    ci_d17 <- c(d17 - t_val * se_d17, d17 + t_val * se_d17)

    # Bootstrap: compute d9 directly from resampled groups (d17 is
deterministic)
    d9_boot <- numeric(n_boot)
    pb <- progress_bar$new(total = n_boot)
    for (i in 1:n_boot) {
      sampled_group <- sample(R, size = N, replace = TRUE, prob = p)
      prop_sample <- table(factor(sampled_group, levels = 1:R)) / N
      sum_p_term_sample <- sum(prop_sample * (1 - prop_sample))

      d9_boot[i] <- rho_gX / sqrt(1 - rho_gX^2) * sqrt(1 /
sum_p_term_sample) * sqrt(4 * (R - 1) / R)
```

```
      pb$tick()
    }
    ci_d9_boot <- quantile(d9_boot, c(0.025, 0.975))

    # Printing
    cat("d8 = d9 (general formula):", round(d9,4), " (",
label_magnitude(d9), ")\n", sep = "")
    cat(" traditional CI95% [", round(ci_d9[1],4), ", ", round(ci_d9[2],4),
"]\n", sep = "")
    cat(" bootstrap CI95% [", format(round(ci_d9_boot[1],3), nsmall=3), ",
", format(round(ci_d9_boot[2],3), nsmall=3), "]\n", sep = "")

    cat("d17 (traditional shortcut):", round(d17,4), " (",
label_magnitude(d17), ")\n", sep = "")
    cat(" traditional CI95% [", round(ci_d17[1],4), ", ",
round(ci_d17[2],4), "]\n", sep = "")
    cat(" bootstrap CI95% not applicable (deterministic)\n")

    return(invisible(list(
      d8 = d9, d17 = d17,
      ci_d8_trad = ci_d9, ci_d8_boot = ci_d9_boot,
      ci_d17_trad = ci_d17
    )))
  }
}

# Activate either case
if (!is.null(RXY) && !is.na(RXY) && is.numeric(RXY)) {
  results_continuous <- compute_d_values(RXY = RXY, p = NULL, N = N, n_boot
= n_boot)
} else if (!is.null(RgX) && !is.null(p) && all(!is.na(p))) {
  results_discrete <- compute_d_values(rho_gX = RgX, p = p, N = N, n_boot =
n_boot)
} else {
  stop("Provide either a valid RXY for continuous case or rho_gX with p for
discrete case.")
}
```
